# Room Temperature Synthesis and Antibacterial Activity of New Sulfonamides Containing *N,N*-Diethyl-Substituted Amido Moieties

**DOI:** 10.1155/2012/367815

**Published:** 2012-10-17

**Authors:** Olayinka O. Ajani, Oluwole B. Familoni, Feipeng Wu, Johnbull O. Echeme, Zheng Sujiang

**Affiliations:** ^1^Department of Chemistry, Covenant University, Canaanland, P.M.B. 1023, Ogun State, Ota, Nigeria; ^2^Department of Chemistry, University of Lagos, Lagos State, Akoka 100001, Nigeria; ^3^New Functional Polymeric Material Group, Technical Institute of Physics and Chemistry, Chinese Academy of Sciences (CAS), Beijing 100190, China; ^4^Test Center of Antimicrobial Materials, Technical Institute of Physics and Chemistry, Chinese Academy of Sciences (CAS), Beijing 100190, China

## Abstract

Sulfonamide drugs which have brought about an antibiotic revolution in medicine are associated with a wide range of biological activities. We have synthesized a series of *α*-tolylsulfonamide, **1–11** and their substituted *N,N*-diethyl-2-(phenylmethylsulfonamido) alkanamide derivatives, **12–22** in improved and excellent yields in aqueous medium at room temperature through highly economical synthetic routes. The chemical structures of the synthesized compounds **1–22** were confirmed by analytical and spectral data such as IR, ^1^H- and ^13^C-NMR, and mass spectra. The *in vitro* antibacterial activity of these compounds along with standard clinical reference, streptomycin, was investigated on two key targeted organisms. It was observed that 1-(benzylsulfonyl)pyrrolidine-2-carboxylic acid, **2** emerged as the most active compound against *Staphylococcus aureus* at MIC value of 1.8 *μ*g/mL while 4-(3-(diethylamino)-3-oxo-2-(phenylmethylsulfonamido) propyl)phenyl phenylmethanesulfonate, **22** was the most active sulfonamide scaffold on *Escherichia coli* at MIC value of 12.5 *μ*g/mL.

## 1. Introduction

The development of sulfonamides is a fascinating and informative area in medicinal chemistry [1–3]. Its functional group has a long and rich history in organic chemistry and drug discovery [4, 5]. The *p*-toluenesulfonamide and benzenesulfonamides have been widely explored in synthetic chemistry [4, 5]; however, few work has been done on the *α*-tolylsulfonamide. For instance, synthesis of benzenesulfonamide derivative of pipecolic acid [6] and that of glycine [7] had been reported. In addition, benzenesulfonamide of alanine was achieved as a result of synthetic usage of such amino acid as a linker to 6*H*-1,3,4-thiadiazine scaffold [8] while naphthylsulfonamide was prepared as antagonist of chemokine receptor [9]. Domagk's discovery of antibacterial activity for the azo dye prontosil led to the first effective chemotherapeutic agent, sulfanilamide [10]. A retrospective look at sulfonamides leaves no doubt that besides providing the first effective treatment of bacterial infections [10, 11], they also unleashed an antibiotic revolution in medicine [12–15] to rationally design new therapeutic agents [16, 17]. These compounds provided an excellent lead for structural modification and ushered in the modern era of chemotherapy and drug design. Sulfonamides inhibit the multiplication of bacteria by acting as competitive inhibitors of *p*-aminobenzoic acid (PABA) in the folic acid metabolism cycle [18, 19]. In fact, the discovery that sulfonamides act through folate inhibition resulted in the development of dihydrofolate reductase inhibitors such as trimethoprim [20, 21]. 

Furthermore, sulfonamide moiety has a crucial functionality because of its wide variety of reported biological [22–25] and pharmacological activities such as anticancer [26–28], carbonic anhydrase inhibitory [29–31], antibacterial [32–34], antimalarial [35, 36], antitumor [37, 38], antihypertensive [39], anti-inflammatory [40–42], and antiprotozoal activities [43]. Some sulfonamides have been established as potent drugs in treatment of insomnia and other sleepless challenges in man by antagonizing orexin neural activity [44–46]. Sulfonamidophenyl porphyrins have a great potential utility as model for activatable photosensitizers [47]. Sulfonamide has also been reported to possess good herbicidal [48] and corrosion inhibitory properties [49, 50]. Multidrug resistance is one of the major immediate threats to human health today [51]. For instance, methicillin is a good antibacterial agent, yet, methicillin resistance among *Staphylococcus aureus* [52] and *Staphylococcus epidermidis* [53, 54] as well as other drug resistance in *Escherichia coli* [55] had been identified to be of great concern in public health. Epidemiological studies have also revealed that the emergent of new diseases is on the increase and quite alarming [56, 57]. 

In addition, the choice of *S. aureus* and *E. coli* as the targeted organisms in this study was due to wide range of infectious diseases and life threatening conditions associated with such isolates.* S. aureus, *which produces heat stable toxin, is among the invasive gram positive known as pyogenic cocci implicated in several diseases of human [58, 59]. From the literatures, *S. aureus *had shown to be very resistant to a wide variety of antibiotics [60]. Infections caused by methicillin-resistant *S. aureus *(MRSA) and vancomycin-resistant *S. aureus *are associated with high morbidity and mortality, high treatment cost, and long stays in hospitals [60]. *E. coli*, a facultative anaerobe of wide distribution in the environment, has been implicated in the cause of urinary tract infections, meningitis, sepsis, wound infections, nosocomial pneumonia, and arthritis. A subgroup enterohemorrhagic *E. coli *(EHEC) can cause severe potentially fatal illness known as hemorrhagic colitis with symptoms of blood diarrhea and severe abdominal pain [61].

Based on the numerous applications of sulfonamides and challenges associated with drug usage and multidrug resistance microorganisms aforementioned among others, there is a continuous need for the synthesis of new organic compounds as potential antimicrobial agents for the replacement of the old existing ones currently available in the market or to enhance the potency of the former ones. Thus, it is conceivable to develop a series of functionalized sulfonamides in order to investigate the *in vitro* antibacterial activities of such scaffolds on the targeted organisms, namely, *S. aureus* and *E. coli.*

## 2. Results and Discussion

### 2.1. Chemistry

**Scheme 1:**
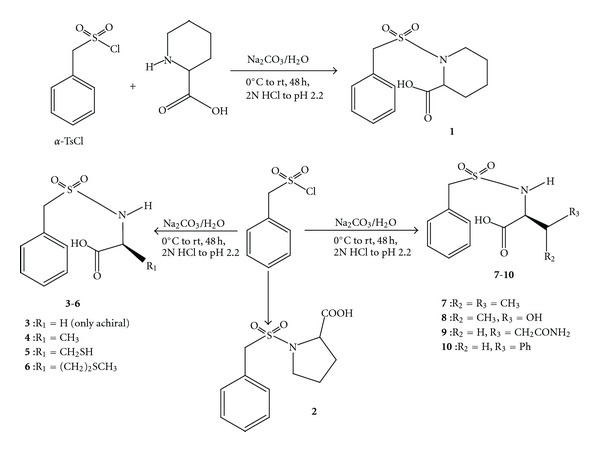
Synthesis of *α*-tolylsulfonamide derivatives **1**–**10**.

In the continuation of our effort on the discovery of rationally designed therapeutic agents [62, 63], we have herein reported the synthesis of a series of new alkylarylsulfonamide with potent antibacterial activity. We started with the reaction of equimolar proportion of readily available amino acid, L-pipecolic acid with *α*-toluene sulfonylchloride (*α*-TsCl) via a continuous magnetic stirring at room temperature in the presence of aqueous sodium carbonate for 48 h according to a known procedure [64]. After the completion of the reaction which was TLC monitored, the excess unreacted *α*-TsCl was recovered with dichloromethane (DCM) using separatory funnel while the aqueous layer was worked up by acidifying to a pH of 2.2 to get clear solution which upon freeze drying and column purification afforded 1-(benzylsulfonyl)piperidine-2-carboxylic acid **1** in excellent yield (98%). Being motivated by this encouraging discovery, we then proceeded on the coupling of nine other amino acids with *α*-TsCl under similar condition to obtain *α*-tolylsulfonamides **2**–**10** in good to excellent yields (Scheme 1). 

**Scheme 2:**
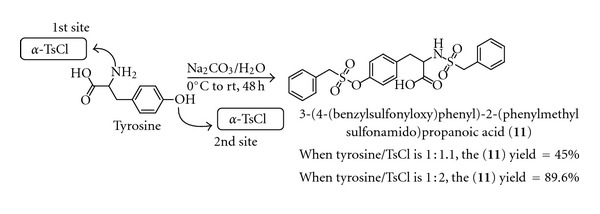
Synthesis of sulfonamide **11** by selective disulfonylation of tyrosine.

In contrast to monosulfonylation that occurred in formation of **1**–**10**, the synthesis of **11** was achieved in higher yield only when disulfonylation of the required amino acid (tyrosine) was utilized. This was noticed when monosulfonylation of tyrosine afforded **11** in lower yield (45%) when compared with other sulfonamide products **1**–**10** with their yields ranging from 87.5% to 98.8%. This was because under the monosulfonylation of tyrosine, the reaction terminated when the *α*-TsCl, being the limiting reagent, has been exhausted living excess of the tyrosine in the aqueous medium. This was as a result of two nucleophilic attacking sites available on the L-tyrosine moiety. This means that the phenolate anion was competing with the amino group in terms of the nucleophilic potential on the *α*-TsCl precursor, thereby resulting in an unusually lower yield (45%). Based on this development, the stoichiometric ratio was revisited in the production of **11** to give allowance for selective disulfonylation of tyrosine. Hence, the reaction for the production of **11** was reconducted in such a way that the molar equivalent of *α*-TsCl was doubled that of tyrosine in order to accommodate nucleophilicity from both NH_2_ and PhO^−^of the latter, then the yield for **11** increased drastically to 89.6% (Scheme 2). Structures of all eleven *α*-tolylsulfonamides were confirmed by spectroscopic means which include IR, mass spectra, ^1^H, and ^13^C NMR as well as the elemental analytical data. Thus, the detail structural elucidation was here-in confirmed using compound **1** as the representative of the *α*-tolylsulfonamide templates. The infrared spectrum of compound **1** gave rise to the absorption bands of OH and carbonyl of carboxylic acid at 3422 and 1736 cm^−1^, respectively. Also, the CH stretching of both aliphatic and aromatic occurred at the vibrational frequency of 2974 and 2822 cm^−1^, respectively. The two IR bands for SO_2_ unit were observed at 1159 and 1238 cm^−1^ as expected, whereas the band accountable for C=C of aromatic was observed at 1603 cm^−1^. The only easily accountable band resulting from a bending vibrational mode at the finger print region was Ar-H which appeared at 700 cm^−1^. 

In the ^1^H-NMR of compound **1**, all the five aromatic protons resonated downfield as a singlet at *δ* 7.52 ppm while the CH_2_ of benzylic group appeared as a two-proton singlet at *δ* 4.28 ppm. Considering the upfield region, it was discovered that CH proton adjacent to COOH resonated as a doublet of doublet at *δ* 4.08–4.02 ppm with coupling constants of 3.44 Hz and 15.12 Hz. This was as a result of the fact that its CH_2_ neighbouring protons were not totally chemically equivalent even though they were attached to the same carbon atom. All other remaining pipecolic protons were well accounted for upfield between *δ* 3.57–3.53 and 1.81–1.68 ppm. The justification for their appearance near TMS scale lies in the fact that they were attached to sp^3^ hybridized centre. Although, the molecular ion peak was not observed, the base peak, however, occurred at *m/z* 178.1 which was as a result of the loss of benzylic radical. Other prominent peaks which were formed as a result of some fragmentation patterns appeared at *m/z* 180.1, 179.1, 165.1, 121.0, 77.0, and 64.0 with the intensities of 55%, 65%, 30%, 42%, 13%, and 31.7%, respectively.

**Scheme 3:**
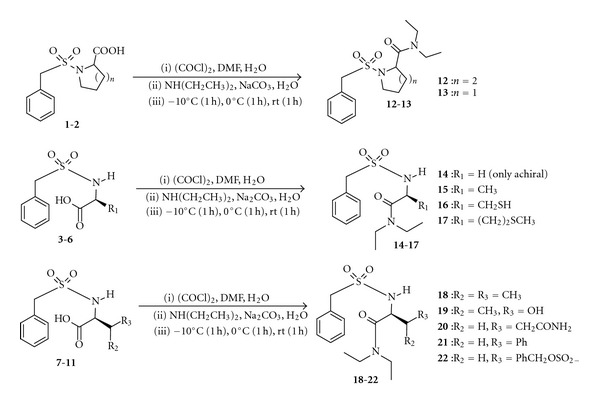
Synthesis of *N*,*N*-diethylamide substituted *α*-tolylsulfonamides, 12–22.

The second stage of this experiment involved the chemical transformation of the carboxylic acid side chain moieties of the sulfonamides **1**–**11** into *N,N*-diethyl alkanamides **12**–**22** using highly efficient nonconventional method in its slightly modified version [65]. The reaction optimization for this aspect was carried out using amination of **1** with diethyl amine under triethyl amine basified condition as a representative. Thus, having established the *C*-amidoalkylation of the sulfonamide **1** to achieve the alkanamide **12** in higher yield as a bountiful success, we were motivated to extend our investigation to the reaction of other *α*-tolylsulfonamides **2**–**11** with diethylamine under the same condition (Scheme 3). These attempts also afforded the *N,N*-diethyl-substituted products **13**–**22** in good to excellent yields as envisaged. The spectroscopic assignment was consistent with that of proposed structures for the obtained *N,N*-diethyl alkanamides **12**–**22**. So, it is necessary to consider the spectral data of **12** as a typical representative of the *N,N*-diethyl alkanamides in order to authenticate such compounds. The IR spectrum of **12** had no absorption band above 3028 cm^−1^ indicating the absence of –OH bond. This in turn confirmed the effective amidation of COOH of compound **1**. The bands at 3028 and 2951 cm^−1^ depicted CH of aromatic and aliphatic, respectively. Also, the C=O and C=C frequency appeared at 1720 and 1593 cm^−1^, respectively, while the two bands of SO_2_ were observed at 1188 and 1148 cm^−1^. Furthermore, the chemical shifts and multiplicity patterns of ^1^H- and ^13^C-NMR correlated well with that of the proposed alkylated sulfonamides **12**–**22**. The ^1^H-NMR spectrum of **12** in D_2_O showed five aromatic protons as singlet at *δ* 7.54 and CH_2_ of benzylic group as singlet at *δ* 4.30. One triplet at *δ* 1.40–1.37 (6H) was assigned to two methyl of diethyl group that were chemically equivalent while one quartet at *δ* 3.21–3.16 (4H) was assigned to two methylene protons adjacent to the methyl protons with a coupling constant of 7.20 Hz. The ^13^C-NMR of **12** in D_2_O in the presence of a drop of dioxane revealed the presence of seventeen different carbon atoms with C=O having the highest signal at *δ* 173.5 ppm while the CH_3_ carbon atoms appeared to have the least signals at *δ* 11.4 ppm.

### 2.2. Antibacterial Activity

 The general antibacterial sensitivity testing (inhibition zone, mm) of all the series of twenty-two synthesized sulfonamides alongside with that of streptomycin clinical standard was assayed on test organisms (*E. coli* and *S. aureus*) using agar diffusion technique [66]. The choice of *E. coli* as the gram −ve organism is because it is easily transmissible through food, water, soil, animal, and man [60]. *E. coli* is a normal flora of human body which causes a lot of vancomycin-resistant *Enterococci* and methicillin-resistant *Staphylococcus aureus* (MRSA) [51]. Based on our previous report [62], the choice of streptomycin as clinical standards is due to the fact that, at low concentrations, streptomycin only inhibits the growth of the bacteria through induction of prokaryotic ribosomes to misread *m*RNA [67] and it also possesses broad spectrum of antibacterial activity. There were reported cases of *E. coli* and *Staphylococcus aureus* being susceptible to streptomycin [4, 68]. The biological relevance of the synthesized sulfonamides here-in was authenticated by screening them *in vitro* against *Staphylococcus aureus* ATCC 6538 (*S. aureus*) and *Escherichia coli* ATCC 25922 (*E. coli*) with the reported selectivity index (SI) duly calculated from zones of inhibition (ZOI). Hence, it should be noted afterwards that the abbreviated forms were given in the bracket for the sake of brevity and conciseness. 

From the result of sensitivity testing, probable activities of *α*-tolylsulfonamide family on the test organisms were categorized based on the size of zone of inhibition (Table 1). Interestingly, it was observed that some of the compounds exhibited probable significant activities based on the large zone of inhibition reported. For instance, compounds **6**, **7**, **12**, **14,** and **22** were highly active on *E*. *coli* while compounds **1**, **2**, **4**, **5**, **10**, **11**, **17,** and **20** exhibited moderate activities on the same organism. All other compounds showed low activities on *E*. *coli* except **3** and **16** which showed no activity at all on the *E*. *coli* even at 1000 *μ*g/mL. The scenario of comparative study of effect of the sulfonamides and streptomycin on *E*. *coli* could be vividly understood by observing the selectivity index (SI). All the sulfonamides have selectivity indices ranging from 0.29 for compound **18** to 0.96 for compound **22** (i.e., less than 1). This implies that streptomycin (SI = 1) was probably more active than any of the sulfonamide scaffolds as regarding the inhibition of *E*. *coli* growth. In the same vein, looking through the effect on *S*. *aureus*, compounds **2**, **5**, **17**, **21,** and **22** were highly active; **4**, **6, 8**, **10**, **11**, **12**, **14,** and **19** were moderately active; **1**, **3**, **7**, **9**, **13**, **15**, **18,** and **20** exhibited low activity while **16** showed no activity on *S*. *aureus *(Table 1). The comparative study of *α*-toluenesulfonamides to streptomycin on *S*. *aureus* growth inhibition is worthy, of commendation. From the SI values, compounds **9**, **12**, **18,** and **20** competed favourably with streptomycin while **2**, **4**, **5**, **6**, **8**, **13**, **14**, **17**, **19**, **21,** and **22** (SI = 1.08–2.31) showed even a better activity than streptomycin on *S*. *aureus*. All other compounds exhibited lesser activity than streptomycin (SI = 0.46–0.92) on *S*. *aureus* except **16** which showed no activity. 

Due to high zones of inhibition obtained during general sensitivity testing, the minimum inhibitory concentration (MIC) was conducted, first at 100 *μ*g/mL using Russell and Furr method [69]. However, those compounds that could not affect the inhibition of microbial growth at this concentration were further repeated for MIC test at 1000 *μ*g/mL. The result of the MIC of this class of compounds on *E*. *coli* and *S*. *aureus* was as shown in Table 2. Interestingly, all the sulfonamides tested showed a concentration-dependent inhibitory effect on the *in vitro* microbial growth assays [70]. Considering the MIC testing on the gram negative organism (*E. coli*), it was observed that compounds** 1, 6, 7, 11, 12, 14,** and **22** inhibited the microbial growth at varying values less than or equal to 100 *μ*g/mL, whereas, all other compounds were active on *E. coli* at higher concentration (between 125 and 1000 *μ*g/mL) except **3** and **16** which had no activity even at 1000 *μ*g/mL. Specifically, MIC values of the synthesized compounds on *E. coli* were reported to be 50 *μ*g/mL for **12** and **14**; 100 *μ*g/mL for **1** and **11**; 125 *μ*g/mL for **2**, **4**, **5**, **10**, **17,** and **20**; 250 *μ*g/mL for **8**, **9**, **13**, **15**, **19,** and **21**. 

 Although, the most active sulfonamides on *E. coli* were **6**, **7,** and **22** with MIC values of 25, 25, and 12.5 *μ*g/mL, respectively, none of them could compete with streptomycin (MIC value of 6.25 *μ*g/mL) in terms of activity. The two rings presence in **22** and their *π* character might be responsible for it being the most active as deduced from the finding of Aissaoui and coworkers [71]. In addition, eight sulfonamides (**2**, **5**, **6**, **8**, **14**, **17**, **21,** and **22**) inhibited the *S*. *aureus* growth at concentration ranging from 1.8 to 100 *μ*g/mL. All other compounds were able to affect the expected inhibition from 125 *μ*g/mL to 1000 *μ*g/mL except **16** which had no activity even at 1000 *μ*g/mL. The significant antibacterial activity of the synthesized compounds may be explained by the ability of its sulfonamide binding site to mimic *p*-aminobenzoic acid (PABA) which is an essential growth factor in the targeted organisms as earlier documented in the literatures [14–16]. The explanation for this encouraging activity of the synthesized sulfonamides could be traceable to the mode of action of sulfonamide drugs. This is based on the inhibition of DNA synthesis [72] by interfering with *para*-aminobenzoic acid (PABA) in biosynthesis of folic acid [73].

Furthermore, from the structure activity relationship (SAR) study, it was observed that the nature of side chains (R_1_ and R_2_-CH-R_3_) of the *α*-tolylsulfonamides and the presence of *N,N*-diethylated amido moieties {(CH_3_CH_2_)_2_N–C=O} of the amide contributed immensely toward synergistic or antagonistic effect on the reported *in vitro* antibacterial activity. For instance, the antibacterial activity of structurally related *α*-tolylsulfonamides containing R_1_ = (CH_2_)_2_SCH_3_, **6**; R_2_ = R_3_ = CH_3_, **7**, on *E. coli* were very high with MIC value of 25 ppm whereas the activity reduced to moderate when R_1_ = CH_3_, **4**; R_1_ = CH_2_SH, **5** with MIC value of 125 ppm. The synergistic effect due to the presence of *N, N*-diethylamido moieties was noticed in some synthetic conversions such as **1** → **12**, **3** → **14** and **11** → **22**. Thus, *α*-tolylsulfonamides **1** and **11** had moderate activity on *E. coli* while their corresponding diethylamido **12** and **22** had better and higher activity on *E. coli*. In fact, *α*-tolylsulfonamide **3** had no activity on *E. coli* whereas its *N, N*-diethyl amidated counterpart, **14** had very high activity with MIC value of 50 ppm. The synergistic effect noticed herein might be as a result of electron donating nature of the R_1_ side chain. On the contrary, antagonistic effect was observed in the synthetic modification of **5** → **16** and **6** → **17** as far as presence of *N, N*-diethyl-substituted amido moieties was concerned. This reverse order of activity on *E. coli* might be as a result of the presence of sulfur atom in **5** and **6** which might have altered the electron donating prowess. Considering the structural relationship effect on the activity of the synthesized compounds against* S. aureus*, it was discovered that *N,N*-diethyl-substituted amides **14**, **17**, **21,** and **22** had better activity than their *α*-tolylsulfonamide precursors **3**,** 6**, **10,** and **11**. 

## 3. Conclusion

It was discovered that aqueous medium approach at ambient temperature used here-in was a highly efficient procedure for the preparation of various *α*-tolylsulfonamide derivatives **1**–**11** in good to excellent yield. The synthetic modification of these derivatives as precursors to furnish disubstituted amide bearing sulfonamides **12**–**22** was also successfully achieved. The antibacterial evaluation study showed 1-(benzylsulfonyl) pyrrolidine-2-carboxylic acid,** 2** to be the most active sulfonamide on *S*. *aureus* while 4-(3-(diethylamino)-3-oxo-2-(phenylmethylsulfonamido)propyl)phenylphenylmethane sulfonate,** 22 **emerged as the most active on the growth inhibition of *E*. *coli*. Thus, this work will be very useful for further studies in terms of toxicity effect and structural activity relationship (SAR) study by using various types of dialkylated substituents to monitor the trend of improvement on their biological and pharmacological properties.

## 4. Experimental

### 4.1. General Conditions

 The ^1^H-NMR spectra were recorded in D_2_O on NMR Bruker DPX 400 spectrometer operating at 400 MHz. TMS was used as an internal standard with the deuterium signal of the solvent as the lock and chemical shifts *δ* recorded in ppm. The ^13^C-NMR spectra were run in addition of a few drops of Dioxane but at 100 MHz frequency. The melting points were determined on XT-4 Digital Binocular Microscope melting point apparatus manufactured by Beijing Technical Instrument Co. Ltd. and were uncorrected. IR spectra were run on Varian Excalibur HE 3100 FT-IR Spectrometer while the mass spectra were obtained using Waters GCT Premier Spectrometer. The elemental analyses (C, H, and N) of the compounds were performed using Flash EA 1112 Elemental Analyzer. Lyophilization was carried out where necessary by using FD-1 Freeze Drier while concentration and removal of solvents were achieved with RE-2000B Rotary Evaporator. 

 In addition, the pH was monitored and confirmed during acidification by using Portable pH Meter Model PHB4. All drying was conducted at a reduced pressure with DHG-9023A Vacuum Oven. The reaction progress was monitored with TLC using CHCl_3_/CH_3_OH solvent system and the developed plates were visualized under UV lamp and/or in iodine tank where necessary. Column chromatographic purifications were carried out on Merck silica gel F (Mesh 200–300). Organic solutions were dried over anhydrous Na_2_SO_4_ and concentrated with a Buchi Rotary Evaporator at reduced pressure. At all stages of the experiments, the synthetic protocols were effected in bone dried solvents under nitrogen atmosphere in dried glassware which were wiped with stream flow of nitrogen gas prior to use and SOCl_2_ was freshly distilled prior to use. Other reagents were used directly after ascertaining the purity condition.

### 4.2. Synthesis

#### 4.2.1. General Procedure of *α*-Tolylsulfonamide Derivatives **1–11**

To a solution of L-amino acid (5 mmol) in H_2_O (6 mL) was added Na_2_CO_3_ (1.113 g, 10.5 mmol) with a continuous stirring until all the solutes had dissolved. The clear solution was cooled to −10°C and *α*-toluenesulfonyl chloride, *α*-TsCl (1.144 g, 6 mmol) was added in three batches over a period of 1 h. It was warmed up to 0°C and stirred there for 1 h. Finally, the reacting mixture was then warmed up to room temperature and allowed to stir there for 48 h. The reaction was quenched by the addition of DCM (10 mL) and transferred into separatory funnel where the excess of *α*-TsCl was removed by extraction. The aqueous layer was then worked up to give a clear solution by the addition of 2N HCl until the pH 2.2 was attained. The clear liquid was then lyophilized at −52°C under reduced pressure for 12 h to obtain the crude solid product which was purified by column chromatography (CHCl_3_/CH_3_OH, 3 : 1) to afford *α*-toluene sulfonamides **1**–**11 **in excellent yields.

**(1) 1-(Benzylsulfonyl)piperidine-2-carboxylic Acid, 1**

Reagents: L-pipecolic acid, yield 98%, mp 248°C (dec), *R*_*f*_ = 0.87. ^1^H-NMR (D_2_O, 400 MHz) *δ*: 7.52 (s, 5H, Ar-H), 4.28 (s, 2H, CH_2_-SO_2_), 4.08–4.02 (dd, *J*_1_ = 3.44 Hz, *J*_2_ = 15.12 Hz, 1H, HOOC-CH-CH_2_), 3.57–3.53 (m, 1H, CHa of CH_2_-N), 3.16–3.10 (m, 1H, CHb of CH_2_-N), 2.40–2.36 (m, 1H, CH), 2.01–1.92 (m, 2H, 2 × CH), 1.81–1.68 (m, 3H, CH & CH_2_) ppm. ^13^C-NMR (Dioxane, 100 MHz) *δ*: 172.5 (CO), 132.5, 131.2 (2CH aromatic), 129.5 (2CH aromatic), 128.9, 57.7, 57.6, 44.8, 26.5, 22.1 (2CH_2_) ppm. IR (KBr) cm^−1^: 3422 (OH of acid), 2974 (CH aromatic), 2822 (CH aliphatic), 1736 (C=O of COOH), 1603 (C=C), 1238, 1159 (SO_2_ two bands), 700 (Ar-H). MS: in m/z [rel. %]: 269.1 [M^+^-CH_2_^*·*^, 3.2%], 180.1 [55%], 179.1 [65%], 178.1 [M^+^-PhCH_2_, 100%], 165.1 [30%], 121.0 [42%], 77.0 [Ph^+^, 13%], 64.0 [SO_2_^+^, 31.7%]. Anal. Calcd. for 283.35 C_13_H_17_NO_4_S: C, 55.11; H, 6.05; N, 4.94. Found: C, 55.29; H, 5.94; N, 4.86.

**(2) 1-(Benzylsulfonyl)pyrrolidine-2-carboxylic Acid, 2**

L-amino acid is L-proline; yield 1.24 g (92%); mp 108–110°C; *R*_*f*_ = 0.84. ^1^H-NMR (D_2_O, 400 MHz) *δ*: 7.47 (s, 5H, Ar-H), 4.46–4.43 (dd, *J*_1_ = 7.2 Hz, *J*_2_ = 15.76 Hz, 1H, HOOC-CH-CH_2_(a,b)), 4.24 (s, 2H, CH_2_-SO_2_), 3.48–3.45 (t, *J* = 7.28 Hz, 2H, N-CH_2_-CH_2_), 2.46-2.45 (m, 1H, CHa of CH_2_), 2.22-2.21 (m, 1H, CHb of CH_2_), 2.15–2.09 (quintet, *J* = 6.8 Hz, 2H, CH_2_-CH_2_-CH_2_(a,b)) ppm. ^13^C-NMR (Dioxane, 100 MHz) *δ*: 173.1 (CO), 132.6, 131.2 (2CH aromatic), 129.5 (2CH aromatic), 128.9, 60.6, 57.7, 47.1, 29.2, 24.3 ppm. IR (KBr) cm^−1^: 3441 (OH of acid), 2980 (CH aromatic), 2828 (CH aliphatic), 1728 (C=O of COOH), 1620 (C=C), 1219, 1151 (SO_2_ two bands), 700 (Ar-H). MS: in *m/z* [rel. %]: 270.1 [MH^+^, 6.5%], 269.1 [M^+^, 9%], 179.1 [18.4%], 178.1 [M^+^-PhCH_2_^*·*^, 100%], 176.1 [32.4%], 122.0 [49%], 105.0 [32%]. Anal. Calcd. for 269.32 C_12_H_15_NO_4_S: C, 53.52; H, 5.61; N, 5.20. Found: C, 53.77; H, 5.49; N, 5.34.

**(3) 2-(Phenylmethylsulfonamido)acetic Acid, 3**

Amino acid is glycine; yield 1.08 g (94%); mp 150-151°C; *R*_*f*_ = 0.51. ^1^H-NMR (D_2_O, 400 MHz) *δ*: 7.45 (s, 5H, Ar-H), 4.19 (s, 2H, CH_2_-SO_2_), 3.76 (s, 2H, CH_2_-COOH) ppm. ^13^C-NMR (Dioxane, 100 MHz) *δ*: 170.6 (CO), 132.7, 131.2 (2CH aromatic), 129.5 (2CH aromatic), 128.9, 57.7, 40.8 ppm. IR (KBr) cm^−1^: 3433 (OH of acid), 3030 (N–H), 2990 (CH aromatic), 2832 (CH aliphatic), 1736 (C=O of COOH), 1616 (C=C), 1215, 1171 (SO_2_ two bands), 702 (Ar-H). MS: in *m/z* [rel. %]: 212.1 [M^+^-OH, 7.9%], 180.1 [73%], 179.1 [88%], 178.1 [M^+^ - PhCH_2_, 100%], 91.1 [PhCH_2_^+^, 48%], 64 [26%] 45 [^+^COOH, 2.4%]. Anal. Calcd. for 229.26 C_9_H_11_NO_4_S: C, 47.15; H, 4.84; N, 6.11. Found: C, 46.90; H, 5.01; N, 5.97.

**(4) 2-(Phenylmethylsulfonamido)propanoic Acid, 4**

L-amino acid is L-alanine; yield 1.18 g (97%); mp 126–128°C; *R*_*f*_ = 0.81. ^1^H-NMR (D_2_O, 400 MHz) *δ*: 7.47 (s, 5H, Ar-H), 4.22 (s, 2H, CH_2_-SO_2_), 4.14–4.08 (q, *J* = 7.28 Hz, 1H, CH-CH_3_), 1.60-1.58 (d, *J* = 7.28 Hz, 3H, CH_3_-CH) ppm. ^13^C-NMR (Dioxane, 100 MHz) *δ*: 176.8 (CO), 131.0, 130.6 (2CH aromatic), 129.3 (2CH aromatic), 129.1, 60.4, 52.1, 19.6 ppm. IR (KBr) cm^−1^: 3424 (OH of acid), 2974 (CH aromatic), 2822 (CH aliphatic), 1751 (C=O of COOH), 1599 (C=C), 1213, 1169 (SO_2_ two bands), 698 (Ar-H). MS: in *m/z* [rel. %]: 212.1 [22%], 180.1 [81.5%], 179.1 [91%], 178.1 [85%], 165.1 [M-PhCH_3_, 55%], 122.0 [80%], 121.0 [100%], 77.0 [Ph^+^, 71.4%], 64.0 [SO_2_^+^, 54.6%], 51.0 [28%]. Anal. Calcd. for 243.28 C_10_H_13_NO_4_S: C, 49.37; H, 5.39; N, 5.76. Found: C, 49.29; H, 5.28; N, 5.94.

**(5) 3-Mercapto-2-(phenylmethylsulfonamido)propanoic Acid, 5**

L-amino acid is cysteine; yield 1.22 g (89%); mp 171–173°C; *R*_*f*_ = 0.49. ^1^H-NMR (D_2_O, 400 MHz) *δ*: 7.49 (s, 5H, Ar-H), 4.50-4.47 (dd, *J*_1_ = 4.24 Hz, *J*_2_ = 7.92 Hz, 1H, CH_2_-CH-COOH), 4.24 (s, 2H, CH_2_-SO_2_), 3.55–3.50 (dd, *J*_1_ = 4.24 Hz, *J*_2_ = 20 Hz, 1H, CHa of CH_2_-CH), 3.39–3.33 (dd, *J*_1_ = 7.92 Hz, *J*_2_ = 20 Hz, 1H, CHb of CH_2_-CH) ppm. ^13^C-NMR (Dioxane, 100 MHz) *δ*: 171.5 (CO), 132.6, 131.3 (2CH aromatic), 129.5 (2CH aromatic), 128.7, 57.8, 52.7, 37.2 ppm. IR (KBr) cm^−1^: 3439 (OH of acid), 2978 (CH aromatic), 2832 (CH aliphatic), 1728 (C=O of COOH), 1618 (C=C), 1207, 1159 (SO_2_ two bands), 810 (Ar-H). MS: in *m/z* [rel. %]: 214.1 [31.7%], 123.0 [100%], 122.0 [90%], 92.1 [PhCH_3_^+^, 33%], 91.0 [PhCH_2_^+^, 88%], 77.0 [Ph^+^, 8%], 65.0 [HSO_2_^+^, 34%], 45.0 [^+^COOH, 28%], 36.0 [34%]. Anal. Calcd. for 275.35 C_10_H_13_NO_4_S_2_: C, 43.62; H, 4.76; N, 5.09. Found: C, 43.45; H, 4.94; N, 5.07.

**(6) 4-(Methylthio)-2-(phenylmethylsulfonamido)butanoic Acid, 6**

L-amino acid is L-methionine; yield 1.33 g (87.7%); mp 89-90°C; *R*_*f*_ = 0.68. ^1^H-NMR (D_2_O, 400 MHz) *δ*: 7.47 (s, 5H, Ar-H), 4.23 (s, 2H, CH_2_-SO_2_), 4.01–3.97 (t, *J* = 8.84 Hz, 1H, CH-CH_2_), 2.76–2.72 (t, *J* = 7.40 Hz, 2H, CH_2_-CH_2_-S), 2.33–2.29 (m, 1H, CH), 2.28–2.21 (m, 1H, CH), 2.19 (s, 3H, CH_3_-S) ppm. ^13^C-NMR (Dioxane, 100 MHz) *δ*: 172.9 (CO), 132.6, 131.2 (2CH aromatic), 129.5 (2CH aromatic), 128.7, 57.9, 52.8, 29.8, 29.4, 14.7 ppm. IR (KBr) cm^−1^: 3442 (OH of acid), 2974 (CH aromatic), 2833 (CH aliphatic), 2774 (CH aliphatic), 1742 (C=O of COOH), 1590 (C=C), 1211, 1161 (SO_2_ two bands), 698 (Ar-H). Anal. Calcd. for 303.40 C_12_H_17_NO_4_S_2_: C, 47.51; H, 5.65; N, 4.62. Found: C, 47.49; H, 5.64; N, 4.66.

**(7) 3-Methyl-2-(phenylmethylsulfonamido)butanoic Acid, 7**

L-amino acid is L-valine; yield 1.34 g (98.7%); mp 137-138°C; *R*_*f*_ = 0.83. ^1^H-NMR (D_2_O, 400 MHz) *δ*: 7.49 (s, 5H, Ar-H), 4.25 (s, 2H, CH_2_-SO_2_), 4.04-4.03 (d, *J *= 4.4 Hz, 1H, CH-CH-COOH), 2.47–2.38 (m, 1H, CH-CH-(CH_3_)_2_), 1.15–1.13 (d, *J* = 7.04 Hz, 3H, CH_3_-CH), 1.13–1.11 (d, *J* = 7.08 Hz, 3H, CH_3_-CH) ppm. ^13^C-NMR (Dioxane, 100 MHz) *δ*: 172.4 (CO), 132.5, 131.2 (2CH aromatic), 129.5 (2CH aromatic), 128.9, 59.2, 57.7, 29.8, 18.1, 17.8 ppm. IR (KBr) cm^−1^: 3447 (OH of acid), 2974 (CH aromatic), 2833 (CH aliphatic), 2783 (CH aliphatic), 1730 (C=O of COOH), 1618 (C=C), 1225, 1165 (SO_2_ two bands), 700 (Ar-H). MS: in *m/z* [rel. %]: 271.1 [M^+^, 14%], 91.0 [PhCH_2_^+^, 30%], 75.0 [65%], 72.1 [100%], 55.0 [79%], 29.0 [50%]. Anal. Calcd. for 271.34 C_12_H_17_NO_4_S: C, 53.12; H, 6.32; N, 5.16. Found: C, 53.31; H, 6.50; N, 5.20.

**(8) 3-Hydroxy-2-(phenylmethylsulfonamido)butanoic Acid, 8**

L-amino acid is L-threonine; yield 1.25 g (91.5%); mp 194-195°C; *R*_*f*_ = 0.48. ^1^H-NMR (D_2_O, 400 MHz) *δ*: 7.49 (s, 5H, Ar-H), 4.48–4.46 (m, 1H, CH), 4.24 (s, 2H, CH_2_-SO_2_), 4.05-4.04 (d, *J* = 4.4 Hz, 1H, CH-CH-COOH), 1.42–1.41 (d, *J* = 6.64 Hz, 3H, CH_3_-CH) ppm. ^13^C-NMR (Dioxane, 100 MHz) *δ*: 171.5 (CO), 132.5, 131.2 (2CH aromatic), 129.5 (2CH aromatic), 128.9, 66.1, 59.4, 57.7, 19.8 ppm. IR (KBr) cm^−1^: 3404 (OH of acid), 2976 (CH aromatic), 2824 (CH aliphatic), 1740 (C=O of COOH), 1601 (C=C), 1219, 1157 (SO_2_ two bands), 700 (Ar-H). Anal. Calcd. for 273.31 C_11_H_15_NO_5_S: C, 48.34; H, 5.53; N, 5.12. Found: C, 48.29; H, 5.61; N, 4.98.

**(9) 5-Amino-5-oxo-2-(phenylmethylsulfonamido)pentanoic Acid, 9**

L-amino acid is L-glutamine; yield 1.41 g (94.1%); mp 211–214°C; *R*_*f*_ = 0.38. ^1^H-NMR (D_2_O, 400 MHz) *δ*: 7.48 (s, 5H, Ar-H), 4.47–4.43 (dd, *J*_1_ = 5.04 Hz, *J*_2_ = 14.32 Hz, 1H, HOOC-CH-CH_2_), 4.23 (s, 2H, CH_2_-SO_2_), 2.62–2.55 (m, 1H, CH), 2.49–2.44 (dd, *J*_1_ = 9.2 Hz, *J*_2_ = 18.72 Hz, 2H, CH_2_-CH_2_-CON), 2.27–2.20 (m, 1H, CH) ppm. ^13^C-NMR (Dioxane, 100 MHz) *δ*: 181.1 (CO of acid), 163.2 (CO of amide), 132.5, 131.2 (2CH aromatic), 129.5 (2CH aromatic), 128.9, 59.2, 57.7, 32.3, 26.2 ppm. IR (KBr) cm^−1^: 3246 (OH of acid), 3075, 3053 (NH two bands), 2983 (CH aromatic), 2951 (CH aliphatic), 1703 (C=O of COOH), 1659 (C=O amide), 1412 (OH bending in-plane), 1221, 1193 (SO_2_ two bands), 696 (Ar-H), 631 (N-H bending with wagging). Anal. Calcd. for 300.34 C_12_H_16_N_2_O_5_S: C, 47.99; H, 5.37; N, 9.33. Found: C, 48.03; H, 5.56; N, 9.41.

**(10) 3-Phenyl-2-(phenylmethylsulfonamido)propanoic Acid, 10**

L-amino acid is L-phenyl alanine; yield = 1.58 g (98.8%); mp 158-159°C (dec); *R*_*f*_ = 0.84. ^1^H-NMR (D_2_O, 400 MHz) *δ*: 7.46 (s, 10H, 2 × Ar-H), 4.34–4.31 (dd, *J*_1_ = 5.60 Hz, *J*_2_ = 13.28 Hz, 1H, PhCH_2_-CH-COOH), 4.22 (s, 2H, CH_2_-SO_2_), 3.41–3.36 (dd, *J*_1_ = 5.60 Hz, *J*_2_ = 20.00 Hz, CHa of CH_2_-Ph), 3.28–3.22 (dd, *J*_1_ = 7.60 Hz, *J*_2_ = 20 Hz, 1H, CHb of CH_2_-Ph) ppm. ^13^C-NMR (Dioxane, 100 MHz) *δ*: 172.3 (CO of acid), 134.8, 132.6, 131.2 (2 × CH aromatic), 130.3 (2 × CH aromatic), 130.1 (2 × CH aromatic), 129.5 (2 × CH aromatic), 128.9 (2 × CH aromatic), 57.7, 55.0, 36.4 ppm. IR (KBr) cm^−1^: 3441 (OH of acid), 2974 (NH), 2822 (CH aromatic), 2776 (CH aliphatic), 1740 (C=O of COOH), 1609 (C=C), 1221, 1171 (SO_2_ two bands), 702 (Ar-H), 623 (N-H bending). MS: in *m/z* [rel. %]: 270.1 [4%], 212.1 [12%], 180.1 [90%], 179.1 [95%], 178.1 [100%], 165.1 [60%], 122.0 [34%], 121.0 [67%], 64 [SO_2_^+^, 70%]. Anal. Calcd. for 319.38 C_16_H_17_NO_4_S: C, 60.17; H, 5.37; N, 4.39. Found: C, 59.98; H, 5.21; N, 4.42.

**(11) 3-(4-(Benzylsulfonyloxy)phenyl)-2-(phenylmethylsulfonamido)propanoic Acid, 11**

L-amino acid is tyrosine; yield = 2.19 g (89.6%); mp 195-196°C; *R*_*f*_ = 0.82. ^1^H-NMR (D_2_O, 400 MHz) *δ*: 7.43 (s, 10H, 2 × Ar-H), 7.22–7.20 (d, *J* = 8.4 Hz, 2H, OTs-H), 6.93–6.91 (d, *J* = 8.4 Hz, 2H, OTs-H), 4.31–4.28 (dd, *J*_1_ = 5.60 Hz, *J*_2_ = 13.08 Hz, 1H, PhCH_2_-CH-COOH), 4.19 (s, 4H, 2 × CH_2_-SO_2_), 3.31-3.26 (dd, *J*_1_ = 5.60 Hz, *J*_2_ = 20.00 Hz, CHa of CH_2_-Ph), 3.19–3.14 (dd, *J*_1_ = 7.52 Hz, *J*_2_ = 20 Hz, 1H, CHb of CH_2_-Ph) ppm. ^13^C-NMR (Dioxane, 100 MHz) *δ*: 172.8 (CO of acid), 156.5, 140.9, 132.0, 131.8 (2CH aromatic), 131.3 (2 × CH aromatic), 129.6 (2 × CH aromatic), 129.0 (2 × CH aromatic), 126.6, 123.6 (2 × CH aromatic), 117.1 (2 × CH aromatic), 111.8 (2 × CH aromatic), 58.4, 55.4, 36.1 ppm. IR (KBr) cm^−1^: 3435 (OH of acid), 3167 (NH), 3028 (CH aromatic), 2949 (CH aliphatic), 1724 (C=O of COOH), 1597 (C=C), 1194, 1148 (SO_2_ two bands), 789 (S-OR ester), 694 (Ar-H), 631 (N-H bending). MS: in *m/z* [rel. %]: 180.1 [74%], 179.1 [90%], 178.1 [100%], 165.1 [30%], 122.0 [10%], 91.1 [PhCH_2_^+^, 47%], 64 [SO_2_^+^, 24%]. Anal. Calcd. for 489.57 C_23_H_23_NO_7_S_2_: C, 56.43; H, 4.76; N, 2.86. Found: C, 56.38; H, 4.79; N, 2.69.

#### 4.2.2. General Procedure of *N,N*-Diethyl-2-(phenylmethylsulfonamido)propanamide,**12–22**

To a solution of *α*-tolylsulfonamide derivatives **1**–**11** (2.96 mmol) in H_2_O (10 mL) in a streaming flow of nitrogen gas was added oxalyl chloride (0.34 mL, 3.85 mmol, 1.30 eq.) via dropping pipette followed by carefully controlled addition of 1 drop of DMF. The resulting mixture was stirred at room temperature for 2 h to get crude acid chloride which was kept air-tighted prior to use. In a separate 250 mL three-necked round bottom flask, equipped with a magnetic stirring bar, was added Na_2_CO_3_ (0.628 g, 5.92 mmol, 2 equiv.) to H_2_O (10 mL) followed by diethyl amine DEA (0.4 mL, 3.85 mmol, 1.3 equiv.) in a continuous stirring and cooled to −15°C. Then, earlier kept acid chloride was added in such a way to maintain the internal temperature of the reaction mixture at around −10°C. The reacting mixture was then stirred at −10°C for 1 h; at 0°C for 1 h; finally at room temperature for 1 h. The reaction was terminated, worked up by acidifying with 2 N HCl, and concentrated in a rotary evaporator. The clear solution obtained was freeze-dried to get crude solid product which was purified by column chromatography (CHCl_3_/CH_3_OH, 3 : 1) to afford *N,N*-dimethylacetamide of *α*-tolylsulfonamide derivatives **12**–**22**.

**(1) 1-(Benzylsulfonyl)-*N,N*-diethylpiperidine-2-carboxamide, 12**

Yield 0.99 g (99.0%); mp 210-211°C; *R*_*f*_ = 0.72. ^1^H-NMR (D_2_O, 400 MHz) *δ*: 7.54 (s, 5H, Ar-H), 4.30 (s, 2H, CH_2_-SO_2_), 4.08–4.04 (dd, *J*_1_ = 3.52 Hz, *J*_2_ = 20.00 Hz, 1H, CHa of CH_2_-N), 3.60–3.57 (m, 1H, CH-CON), 3.21–3.16 (q, *J* = 7.20 Hz, 4H, 2 × CH_2_-CH_3_), 2.42–2.38 (dd, *J*_1_ = 3.32 Hz, *J*_2_ = 20.00 Hz, 1H, CHb of CH_2_-N), 2.03–1.99 (m, 2H, CH_2_), 1.86–1.73 (m, 3H, CH & CH_2_), 1.40–1.37 (t, *J* = 7.20 Hz, 6H, 2 × CH_3_-CH_2_) ppm. ^13^C-NMR (Dioxane, 100 MHz) *δ*: 173.5 (C=O), 132.9, 131.1 (2 × CH aromatic), 129.8 (2 × CH aromatic), 128.6, 60.6, 57.8, 47.4, 43.4 (CH_2_), 29.4, 24.2, 18.1, 11.4 (CH_3_) ppm. IR (KBr) cm^−1^: 3028 (CH aromatic), 2951 (CH aliphatic), 1720 (C=O), 1593 (C=C), 1188, 1148 (SO_2_ two bands), 696 (Ar-H). Anal. Calcd. for C_17_H_26_N_2_O_3_S (338.47): C, 60.33; H, 7.74; N, 8.28. Found: C, 60.29; H, 6.94; N, 7.98.

**(2) 1-(Benzylsulfonyl)-*N,N*-diethylpyrrolidine-2-carboxamide, 13**

Yield 0.94 g (97.9%); mp 185–187°C; *R*_*f*_ = 0.71. ^1^H-NMR (D_2_O, 400 MHz) *δ*: 7.53 (s, 5H, Ar-H), 4.54–4.50 (m, 1H, CH-CON), 4.28 (s, 2H, CH_2_-SO_2_), 3.56–3.54 (m, 2H, CH_2_-N), 3.19–3.14 (q, *J* = 7.28 Hz, 4H, 2 × CH_2_-CH_3_), 2.54–2.51 (m, 1H, CH), 2.28–2.24 (m, 1H, CH), 2.16–2.14 (m, 2H, CH_2_), 1.39–1.35 (t, *J* = 7.28 Hz, 6H, 2 × CH_3_-CH_2_) ppm. ^13^C-NMR (Dioxane, 100 MHz) *δ*: 173.2 (C=O), 132.6, 131.1 (2 × CH aromatic), 129.4 (2 × CH aromatic), 128.8, 60.8, 57.7, 47.1, 43.3 (2 × CH_2_), 29.2, 24.2, 11.4 (2 × CH_3_) ppm. Anal. Calcd. for C_16_H_24_N_2_O_3_S (324.45): C, 59.23; H, 7.46; N, 8.63. Found: C, 59.09; H, 7.46; N, 8.48.

**(3) *N,N*-Diethyl-2-(phenylmethylsulfonamido)acetamide, 14**

Yield 0.78 g (92.6%); mp 213–215°C; *R*_*f*_ = 0.51. ^1^H-NMR (D_2_O, 400 MHz) *δ*: 7.53 (s, 5H, Ar-H), 4.29 (s, 2H, CH_2_-SO_2_), 4.01 (s, 2H, CH_2_-CON), 3.20-3.14 (q, *J* = 7.30 Hz, 4H, 2 × CH_2_-CH_3_), 1.39–1.36 (t, *J* = 7.30 Hz, 6H, 2 × CH_3_-CH_2_) ppm. ^13^C-NMR (Dioxane, 100 MHz) *δ*: 174.1 (C=O), 133.4, 131.6 (2 × CH aromatic), 129.5 (2 × CH aromatic), 128.6, 57.7, 49.8, 43.1 (CH_2_), 11.4 (CH_3_) ppm. IR (KBr) cm^−1^: 3217 (N-H), 3036 (CH aromatic), 2947 (CH aliphatic), 1712 (C=O), 1601 (C=C), 1219, 1194, (SO_2_ two bands), 694 (Ar-H). Anal. Calcd. for C_13_H_20_N_2_O_3_S (284.38): C, 54.91; H, 7.09; N, 9.85. Found: C, 55.13; H, 6.94; N, 10.08.

**(4) *N,N*-Diethyl-2-(phenylmethylsulfonamido)propanamide, 15**

Yield 0.87 g (98.5%); mp 238–240°C; *R*_*f*_ = 0.56. ^1^H-NMR (D_2_O, 400 MHz) *δ*: 7.50 (s, 5H, Ar-H), 4.26 (s, 2H, CH_2_-SO_2_), 4.22–4.16 (q, *J* = 7.28 Hz, 1H, CH-CH_3_), 3.18–3.12 (q, *J* = 7.32 Hz, 4H, 2 × CH_2_-CH_3_), 1.65–1.63 (d, *J* = 7.28 Hz, 3H, CH_3_-CH), 1.37–1.33 (t, *J *= 7.32 Hz, 6H, 2 × CH_3_-CH_2_) ppm. ^13^C-NMR (Dioxane, 100 MHz) *δ*: 173.8 (C=O), 132.7, 131.2 (2 × CH aromatic), 129.5 (2 × CH aromatic), 128.9, 57.7, 49.8, 43.1 (CH_2_), 16.2, 11.4 (CH_3_) ppm. IR (KBr) cm^−1^: 3058 (N-H), 3036 (CH aromatic), 2951 (CH aliphatic), 1719 (C=O), 1601 (C=C), 1219, 1196, 1148 (SO_2_ two bands), 696 (Ar-H). Anal. Calcd. for C_14_H_22_N_2_O_3_S (298.41): C, 56.35; H, 7.43; N, 9.39. Found: C, 56.11; H, 7.33; N, 9.28.

**(5) *N,N*-Diethyl-3-mercapto-2-(phenylmethylsulfonamido)propanamide, 16**

Yield 0.87 g (89.0%); mp 198–200°C; *R*_*f*_ = 0.71. ^1^H-NMR (D_2_O, 400 MHz) *δ*: 7.46 (s, 5H, Ar-H), 4.54–4.51 (dd, *J*_1_ = 4.24 Hz, *J*_2_ = 7.92 Hz, 1H, CH_2_-CH-COOH), 4.21 (s, 2H, CH_2_-SO_2_), 3.57–3.52 (dd, *J*_1_ = 4.24 Hz, *J*_2_ = 20.00 Hz, 1H, CHa of CH_2_-CH), 3.41–3.36 (dd, *J*_1_ = 7.92 Hz, *J*_2_ = 20.00 Hz, 1H, CHb of CH_2_-CH), 3.18–3.12 (q, *J* = 7.35 Hz, 4H, 2 × CH_2_-CH_3_), 1.37–1.33 (t, *J* = 7.35 Hz, 6H, 2 × CH_3_-CH_2_) ppm. ^13^C-NMR (Dioxane, 100 MHz) *δ*: 173.5 (C=O), 132.7, 131.2 (2 × CH aromatic), 129.5 (2 × CH aromatic), 128.9, 57.4, 49.5, 43.1 (CH_2_), 35.5, 11.4 (CH_3_) ppm. IR (KBr) cm^−1^: 2997 (CH aromatic), 2911 (CH aliphatic), 1719 (C=O), 1591 (C=C), 1194, 1144 (SO_2_ two bands), 696 (Ar-H). Anal. Calcd. for C_14_H_22_N_2_O_3_S_2_ (330.47): C, 50.88; H, 6.71; N, 8.48. Found: C, 50.71; H, 6.99; N, 7.97.

**(6) *N,N*-Diethyl-4-(methylthio)-2-(phenylmethylsulfonamido)butanamide, 17**

Yield 0.96 g (90.6%); mp 170–172°C; *R*_*f*_ = 0.65. ^1^H-NMR (D_2_O, 400 MHz) *δ*: 7.49 (s, 5H, Ar-H), 4.24 (s, 2H, CH_2_-SO_2_), 4.23–4.20 (t, *J* = 6.72 Hz, 1H, CH-CH_2_), 3.16–3.10 (q, *J* = 7.32 Hz, 4H, 2 × CH_2_-CH_3_), 2.77–2.73 (t, *J* = 7.40 Hz, 2H, S-CH_2_-CH_2_), 2.34–2.24 (m, 2H, CH-CH_2_-CH_2_-S), 2.19 (s, 3H, CH_3_-S), 1.35–1.31 (t, *J *= 7.32 Hz, 6H, 2 × CH_3_-CH_2_) ppm. ^13^C-NMR (Dioxane, 100 MHz) *δ*: 173.8, 132.7, 131.2 (2 × CH aromatic), 129.5, (2 × CH aromatic), 57.7, 49.8, 43.1 (CH_2_), 30.8, 29.5, 16.2, 11.4 (CH_3_) ppm. IR (KBr) cm^−1^: 3028 (CH aromatic), 2945 (CH aliphatic), 1722 (C=O), 1620 (C=C), 1200, 1126 (SO_2_ two bands) 698 (Ar-H). Anal. Calcd. for C_16_H_26_N_2_O_3_S_2_ (358.53): C, 53.60; H, 7.31; N, 7.81. Found: C, 53.55; H, 7.22; N, 7.69.

**(7) *N,N*-Diethyl-3-methyl-2-(phenylmethylsulfonamido)butanamide, 18**

Yield 0.94 g (97.3%); mp 226–230°C; *R*_*f*_ = 0.69. ^1^H-NMR (D_2_O, 400 MHz) *δ*: 7.50 (s, 5H, Ar-H), 4.25 (s, 2H, CH_2_-SO_2_), 4.00-3.97 (d, *J* = 2.84 Hz, 1H, CH-CH-CON), 3.17–3.11 (q, *J* = 7.32 Hz, 4H, 2 × CH_2_-CH_3_), 2.45–2.38 (m, 1H, CH), 1.36–1.32 (t, *J* = 7.32 Hz, 6H, 2 × CH_3_-CH_2_), 1.15–1.13 (d, *J* = 7.00 Hz, 3H, CH_3_-CH), 1.12–1.10 (d, *J* = 7.00 Hz, 3H, CH_3_-CH) ppm. ^13^C-NMR (Dioxane, 100 MHz) *δ*: 173.8 (C=O), 132.7, 131.2 (2 × CH aromatic), 129.5 (2 × CH aromatic), 128.9, 60.8, 57.7, 49.8, 43.1 (CH_2_), 16.2, 15.5, 11.4 (CH_3_) ppm. IR (KBr) cm^−1^: 3053 (CH aromatic), 2945 (CH aliphatic), 1718 (C=O), 1611 (C=C), 1219, 1194 (SO_2_ two bands), 696 (Ar-H). Anal. Calcd. for C_16_H_26_N_2_O_3_S (326.46): C, 58.87; H, 8.03; N, 8.58. Found: C, 59.01; H, 7.96; N, 8.61.

**(8) *N,N*-Diethyl-3-hydroxy-2-(phenylmethylsulfonamido)butanamide, 19**

Yield 0.78 g (80.2%); mp 245°C (dec); *R*_*f*_ = 0.53. ^1^H-NMR (D_2_O, 400 MHz) *δ*: 7.55 (s, 5H, Ar-H), 4.54–4.52 (m, 1H, CH-CH-CH_3_), 4.31 (s, 2H, CH_2_-SO_2_), 4.09–4.08 (d, *J* = 3.96 Hz, 1H, CH-CH-CON), 3.22–3.17 (q, *J* = 7.32 Hz, 4H, 2 × CH_2_-CH_3_), 2.85 (s, 1H, OH), 1.49–1.47 (d, *J* = 6.60 Hz, 3H, CH_3_-CH), 1.41–1.37 (t, *J* = 7.32 Hz, 6H, 2 × CH_3_-CH_2_) ppm. ^13^C-NMR (Dioxane, 100 MHz) *δ*: 173.5 (C=O), 132.9, 131.3 (2 × CH aromatic), 129.5 (2 × CH aromatic), 128.9, 61.2, 58.1, 49.5, 42.5 (CH_2_), 16.6, 11.1 (CH_3_) ppm. IR (KBr) cm^−1^: 3396 (OH), 3030 (CH aromatic), 2945 (CH aliphatic), 1720 (C=O), 1601 (C=C), 1221, 1194 (SO_2_ two bands), 694 (Ar-H). Anal. Calcd. for C_15_H_24_N_2_O_4_S (328.43): C, 54.86; H, 7.37; N, 8.53. Found: C, 54.71; H, 7.26; N, 8.65.

**(9) *N* ^1^, *N* ^1^ *-*Diethyl-2-(phenylmethylsulfonamido)pentanedi-amide, 20**

Yield 0.98 g (93.2%); mp 251–253°C; *R*_*f*_ = 0.58. ^1^H-NMR (D_2_O, 400 MHz) *δ*: 7.51 (s, 5H, Ar-H), 4.52–4.48 (dd, *J*_1_ = 5 Hz, *J*_2_ = 14.32 Hz, 1H, NOC-CH-CH_2a,b_), 4.26 (s, 2H, CH_2_-SO_2_), 3.17–3.12 (q, *J* = 7.32 Hz, 4H, 2 × CH_2_-CH_3_), 2.67–2.60 (m, 1H, CH_a_ of CH_2_), 2.53–2.48 (t, *J* = 8 Hz, 2H, CO-CH_2_-CH_2_), 2.30–2.24 (m, 1H, CH_b_ of CH_2_), 1.37–1.33 (t, *J* = 7.32 Hz, 6H, 2 × CH_3_-CH_2_) ppm. ^13^C-NMR (Dioxane, 100 MHz) *δ*: 182.7 (C=O), 177.1 (C=O), 132.6, 131.2 (2 × CH aromatic), 129.5 (2 × CH aromatic), 128.9, 57.7, 53.3, 43.1 (CH_2_), 30.1, 25.2, 11.4 (CH_3_) ppm. IR (KBr) cm^−1^: 3075 (NH), 3053 (CH aromatic), 2951 (CH aliphatic), 1703 (C=O), 1659 (C=O of CON), 1601 (C=C), 1221, 1193 (SO_2_ two bands), 696 (Ar-H). Anal. Calcd. for C_16_H_25_N_3_O_4_S (355.46): C, 54.06; H, 7.09; N, 11.82. Found: C, 53.95; H, 6.88; N, 12.01.

**(10) *N,N*-Diethyl-3-phenyl-2-(phenylmethylsulfonamido)propanamide, 21**

Yield 1.00 g (90.2%); mp 227–229°C; *R*_*f*_ = 0.70. ^1^H-NMR (D_2_O, 400 MHz) *δ*: 7.49 (s, 10H, 2 × Ar-H), 4.39–4.36 (dd, *J*_1_ = 5.60 Hz, *J*_2_ = 7.60 Hz, 1H, NOC-CH-CH_2a,b_), 4.24 (s, 2H, CH_2_-SO_2_), 3.44–3.39 (dd, *J*_1_ = 5.60 Hz, *J*_2_ = 20.00 Hz, 1H, CH_a_ of CH_2a,b_), 3.31–3.26 (dd, *J*_1_ = 7.60 Hz, *J*_2_ = 20.00 Hz, 1H, CH_b_ of CH_2a,b_), 3.16–3.10 (q, *J *= 7.32 Hz, 4H, 2 × CH_2_-CH_3_), 2.78 (s, 2H, CH_2_), 1.35–1.31 (t, *J* = 7.32 Hz, 6H, 2 × CH_3_-CH_2_) ppm. ^13^C-NMR (Dioxane, 100 MHz) *δ*: 172.4 (C=O), 135.1, 132.7, 131.2 (2 × CH aromatic), 130.3 (2 × CH aromatic), 130.1 (2 × CH aromatic), 129.5 (2 × CH aromatic), 128.9 (2 × CH aromatic), 57.8, 55.2, 43.1 (CH_2_), 36.5, 11.5 (CH_3_) ppm. IR (KBr) cm^−1^: 2976 (NH), 2828 (CH aromatic), 2774 (CH aliphatic), 1736 (C=O), 1620 (C=C), 1206, 1153, 1148 (SO_2_ two bands), 698 (Ar-H). Anal. Calcd. for C_20_H_26_N_2_O_3_S (374.51): C, 64.14; H, 7.00; N, 7.48. Found: C, 64.00; H, 6.84; N, 7.29.

**(11) 4-(3-(Diethylamino)-3-oxo-2-(phenylmethylsulfonamido)propyl)phenyl phenyl methanesulfonate, 22**

Yield 1.44 g (89.3%); mp 265°C (dec); *R*_*f*_ = 0.69. ^1^H-NMR (D_2_O, 400 MHz) *δ*: 7.50 (s, 10H, 2 × Ar-H), 7.29–7.27 (d, *J* = 8.00 Hz, 2H, Ar-H), 6.99–6.97 (d, *J* = 8.00 Hz, 2H, Ar-H), 4.37–4.33 (dd, *J*_1_ = 5.60 Hz, *J*_2_ = 7.60 Hz, 1H, NOC-CH-CH_2a,b_), 4.26 (s, 2H, CH_2_-SO_2_), 3.37–3.32 (dd, *J*_1_ = 5.60 Hz, *J*_2_ = 20.00 Hz, 1H, CH_a_ of CH_2a,b_), 3.26–3.21 (dd, *J*_1_ = 7.60 Hz, *J*_2_ = 20.00 Hz, 1H, CH_b_ of CH_2a,b_), 3.17–3.12 (q, *J* = 7.32 Hz, 4H, 2 × CH_2_-CH_3_), 1.37–1.33 (t, *J* = 7.32 Hz, 6H, 2 × CH_3_-CH_2_) ppm. ^13^C-NMR (Dioxane, 100 MHz) *δ*: 172.4 (C=O), 155.9, 141.3, 131.8 (2 × CH aromatic), 131.3 (2 × CH aromatic), 129.6 (2 × CH aromatic), 129.0 (2 × CH aromatic), 126.6, 123.0 (2 × CH aromatic), 116.9 (2 × CH aromatic), 111.6 (2 × CH aromatic), 57.8, 55.2, 43.2 (CH_2_), 35.6, 11.6 (CH_3_) ppm. IR (KBr) cm^−1^: 3058 (NH), 3036 (CH aromatic), 2951 (CH aliphatic), 1719 (C=O), 1601 (C=C), 1219, 1196, 1148 (SO_2_ two bands), 696 (Ar-H). Anal. Calcd. for C_27_H_32_N_2_O_6_S_2_ (544.69): C, 59.54; H, 5.92; N, 5.14. Found: C, 59.43; H, 5.99; N, 4.98.

### 4.3. Antibacterial Activity Assays

The antimicrobial properties of the sulfonamides were investigated in the form of the general sensitivity testing and minimum inhibitory concentration (MIC) with respect to freshly cultured targeted organisms. The two organisms of interest in this present study are one gram positive (*Staphylococcus aureus* ATCC 6538) and one gram negative (*Escherichia coli* ATCC 25922) organisms which are associated with the gastrointestinal tract damage in man and animal. 

#### 4.3.1. Preparation of the Inoculum

The standard strains of *S*. *aureus* and *E. coli *used were obtained from Test Center of Antimicrobial Materials, TIPC, Beijing. No clinically isolated organism was used based on inavailability of such as at the time of this study. The strains were propagated on nutrient agar plates and maintained on the plate at 4°C. The isolates were subcultured in nutrient broth at 37°C for 8 h prior to antibacterial testing.

#### 4.3.2. Antibacterial Sensitivity Testing of the Synthesized Compounds

Agar well diffusion technique as described by Adeniyi and coworkers was used to determine the antibacterial activity of the synthesized compounds [66]. Sensitivity test agar plates were seeded with 0.1 mL of an overnight culture of each bacterial strain (equivalent to 10^7^–10^8^ CFU mL^−1^). The seeded plates were allowed to set and a standard cork borer of 8 mm diameter was used to cut uniform wells on the surface of the agar. The wells were then filled with 0.3 mL of each sulfonamide solution in an appropriate solvent at a concentration of 1000 *μ*g/mL (0.02 g of sulfonamide dissolved in 20 mL distilled water). All the plates were incubated at 37°C for 24 h. The assay was conducted at regular intervals of 24 h until a marked decline in the potency of the sulfonamide solution to inhibit the growth of the test organisms was noticed. Zones of clearance round each well means inhibition and the diameter of such zones were measured. The procedure was repeated for the streptomycin (standard).

#### 4.3.3. Determination of Minimum Inhibitory Concentration (MIC)

Agar well dilution method as described by Russell and Furr was used to determine the minimum inhibitory concentration (MIC) of the sulfonamides and streptomycin [69]. Different dilutions of the sulfonamides were prepared first at ≤100 *μ*g/mL to give final concentrations in the range of 100, 50, 25, 12.5, 6.25, and 1.8 *μ*g/mL. The different dilutions of sulfonamide derivatives that could not inhibit the microbial growth at ≤100 *μ*g/mL were later prepared at ≤1000 *μ*g/mL to give final concentrations in the range of 1000, 500, 250, 125, and 62.5 *μ*g/mL. Two milliliter (2 mL) of each dilution was mixed with 18 mL of Mueller Hinton agar (MHA, Difco, France) and poured into Petri dishes and allowed to set. The agar was streaked with an overnight broth culture of the bacterial strains and incubated overnight. The plates were then examined for the presence or absence of growth. The minimum concentration that completely inhibited macroscopic growth was regarded as the minimum inhibitory concentration of the respective sulfonamide. The procedure was repeated for streptomycin (standard). Selectivity index (SI) is the ratio of the zone of the inhibition of compound to that of the streptomycin.

## Figures and Tables

**Table 1:** Result of general sensitivity testing with zones of inhibitory in (mm) and selectivity index.

Compd. no.	*In vitro* antibacterial activity			
	Organisms			
	*E. coli* ATCC 25922		* S. aureus *ATCC 6538	
	ZOI (mm)	SI	ZOI (mm)	SI
**1**	++	0.61	+	0.92
**2**	++	0.54	+++	2.31
**3**	−	—	+	0.69
**4**	++	0.57	++	1.08
**5**	++	0.57	+++	2.15
**6**	+++	0.86	++	1.15
**7**	+++	0.89	+	0.46
**8**	+	0.46	++	1.15
**9**	+	0.36	+	1.00
**10**	++	0.57	++	0.92
**11**	++	0.75	++	0.92
**12**	+++	0.84	++	1.00
**13**	+	0.43	+	1.08
**14**	+++	0.82	++	1.38
**15**	+	0.36	+	0.92
**16**	−	—	−	—
**17**	++	0.57	+++	2.23
**18**	+	0.29	+	1.00
**19**	+	0.43	++	1.08
**20**	++	0.54	+	1.00
**21**	+	0.39	+++	2.00
**22**	+++	0.96	+++	2.00
**Str.**	+++	1.00	++	1.00

+: Less active 5–12 mm; ++: moderately active 13–19 mm; +++: highly active 20–31 mm; −: resistance; str.: streptomycin clinical reference; Z.O.I.: zone of inhibition; S.I.: selective index obtained by comparing inhibition zone of compound to that of streptomycin standard; *E. coli*: *Escherichia coli* (ATCC 25922)^G−^; *S*. *aureus*: *Staphylococcus aureus* (ATCC 6538)^G+^; G−: gram negative; G+: gram positive.

**Table 2:** Result of MIC test of *α*-tolylsulfonamide on targeted organisms (*μ*g/mL).

Compd. no.	Minimum inhibitory concentration (*μ*g/mL)			
	Organisms			
	* E. coli* ATCC 25922		* S. aureus *ATCC 6538	
	at 100 *μ*g/mL	at 1000 *μ*g/mL	at 100 *μ*g/mL	at 1000 *μ*g/mL
**1**	100	<1000	>100	250
**2**	>100	125	1.8	<1000
**3**	>100	—	>100	250
**4**	>100	125	>100	125
**5**	>100	125	50	<1000
**6**	25	<1000	100	<1000
**7**	25	<1000	>100	500
**8**	>100	250	100	<1000
**9**	>100	250	>100	250
**10**	>100	125	>100	125
**11**	100	<1000	>100	125
**12**	50	<1000	>100	125
**13**	>100	250	>100	250
**14**	50	<1000	62.5	<1000
**15**	>100	250	>100	1000
**16**	>100	—	>100	—
**17**	>100	125	25	<1000
**18**	>100	500	>100	1000
**19**	>100	250	>100	125
**20**	>100	125	>100	250
**21**	>100	250	25	<1000
**22**	12.5	<1000	25	<1000
**Str.**	6.25	<1000	>100	125

>100 means that if there was no growth inhibition at 100 μg/mL, it was repeated at 1000 μg/mL, <1000 μg/mL means that growth inhibition has already been experienced at lower concentration less than or equal to 100 μg/mL; hence, there is no need to repeat the test at 1000 μg/mL. — means no activity was observed even at 1000 μg/mL. Str. means streptomycin clinical reference.
